# Understanding the influence of physical resources and social supports on primary food providers’ snack food provision: a discrete choice experiment

**DOI:** 10.1186/s12966-020-01062-y

**Published:** 2020-11-30

**Authors:** Brittany J. Johnson, Rebecca K. Golley, Dorota Zarnowiecki, Gilly A. Hendrie, Elisabeth K. Huynh

**Affiliations:** 1 Caring Futures Institute, College of Nursing & Health Sciences, Flinders University, GPO Box 2100, Adelaide, South Australia 5001 Australia; 2 Early Prevention of Obesity in Childhood Centre for Research Excellence, Sydney, New South Wales Australia; 3 Health & Biosecurity Flagship, Commonwealth Scientific Industrial Research Organisation, Adelaide, South Australia Australia; 4 Department of Health Services Research and Policy, Research School of Population Health, Australian National University, Acton, Australian Capital Territory Australia

**Keywords:** Primary food providers, Snacks, Discrete choice experiment, Decision making, Unhealthy foods, Young children, Food choice, Opportunity

## Abstract

**Background:**

Snack eating occasions contribute approximately a third of children’s energy intake, with approximately half of all unhealthy foods consumed during snack times. Therefore, it is critical to understand the drivers of primary food providers’ snack provision. The study aims were to determine the relative importance of physical resources and social supports when primary food providers are choosing snacks to provide to their child, and to investigate how these attributes differ in social versus non-social occasions, and between subgroups of primary food providers based on socio-economic position.

**Methods:**

Primary food providers of three to seven-year olds completed an online discrete choice experiment, by making trade-offs when completing repeated, hypothetical choice tasks on the choice of snacks to provide to their child in: 1) non-social and 2) social condition. Choice tasks included two alternatives consisting of varying attribute (i.e. factor) levels, and an opt-out option. The order of conditions shown were randomized across participants. Multinomial logit model analyses were used to determine utility weights for each attribute.

**Results:**

Two-hundred and twenty-five primary food providers completed the study, providing 1125 choice decisions per condition. In the non-social condition, the top three ranked attributes were type of food (utility weight 1.94, *p* < 0.001), child resistance (− 1.62, *p* < 0.001) and co-parent support (0.99, *p* < 0.001). In the social condition, top ranking attributes were child resistance (utility weight − 1.50, *p* < 0.001), type of food (1.38, *p* < 0.001) and co-parent support (1.07, *p* < 0.001). In both conditions, time was not a significant influence and cost was of lowest relative importance. Subgroup analyses revealed cost was not a significant influence for families from higher socio-economic backgrounds.

**Conclusions:**

Type of food, child resistance and co-parent support were of greatest relative importance in primary food providers’ snack provision decision-making, regardless of social condition or socio-economic position. In designing future interventions to reduce unhealthy snacks, researchers should prioritize these influences, to better support primary food providers in changing their physical and social opportunity.

**Trial registration:**

Australian New Zealand Clinical Trials Registry no. ACTR N12618001173280

**Supplementary Information:**

The online version contains supplementary material available at 10.1186/s12966-020-01062-y.

## Background

Snack occasions refers to eating occasions that fall outside of the main meals of breakfast, lunch and the evening meal [1]. In Australia and the United States, snacks as an eating occasion contribute approximately 30% of children’s daily energy intake [2, 3]. Additionally, half of Australian children’s unhealthy (energy-dense, nutrient-poor) foods and beverages intake is consumed during snack eating occasions [4] . Improving children’s food intake at snack occasions offers one approach to reducing children’s unhealthy food and beverage intake and enhancing diet quality [5]. As parents are key gatekeepers of children’s food intake [6], understanding factors influencing their food provision choices can provide insights to change children’s intake at snack occasions.

Food choice is complex. It is estimated that adults make over 200 decisions about food everyday [7]. In addition to their own food choices, parents make additional decisions about the food and beverages to provide to their children. Many environmental factors influence food provision, such as the physical resources and social supports that prompt or inhibit food provision [8]. Physical resources such as cost, time, convenience and food availability have been reported as barriers to primary food providers providing healthy food choices to children [9–15]. Additionally, a recent review found young children’s access to unhealthy foods was commonsly associated with higher children’s snack intake, highlighting the importance of availability of snacks [16]. Social factors have also been identified to influence parental food provision including child resistance, requests and preferences [11, 12, 14, 15, 17–19], and grandparents, friends or partners undermining provision choices [9, 11, 14, 20, 21]. The evidence-base to date is informative in that it provides a list of factors that are important for primary food providers yet does not differentiate their level of importance. In addition, many physical or social factors have not been explored together and compared within a single sample of parents. Few studies have explored the influence of physical resources and social supports in primary food providers’ food choice decision-making processes [22–24].

Various contextual factors may also impact parents’ food provision decision-making. For example, differences in children’s intake have been reported when comparing contextual factors such as weekdays and weekends and social context [14, 25]. Our prior research found parents rated several motivational constructs lower in contexts involving visitors or extended family members, compared with contexts involving immediate family [26]. Qualitative research has reported differences in parent reported factors influencing unhealthy food provision when interviewing parents experiencing low versus high socio-economic circumstances [14]. The limited available literature suggests social contexts and socio-economic position may influence parents’ food choice and warrant further exploration when investigating primary food providers’ food choice decision-making processes.

Methodological approaches used to date for understanding decision-making in parental food provision rely on parents’ report, either via qualitative explorations (e.g. [14, 27]) or questionnaires (e.g. [22–24]), and do not determine the relative importance of important factors. Alternative methodologies are required to understand the importance of such factors in parent unhealthy food provision to advance this research area and overcome limitations of past designs. Discrete choice experiments provide an approach to understand the complexity of decision-making by mimicking real world decision-making [28]. This includes forcing participants to make compromises (trade-offs) when making decisions in hypothetical but realistic situations [29]. Discrete choice experiment methodology makes use of choices rooted in real life that provide testable predictions [29]. Although discrete choice experiments have been utilized in the health area for the past 20–30 years [30] they have only emerged in the nutrition field in the last 5 years (e.g. [31]). This study aimed to determine the relative importance of physical resources and social supports when primary food providers are choosing snack foods to provide to their child using discrete choice experiments. Secondary aims were to investigate how the relative importance of physical resources and social supports differ in social versus non-social occasions, and between subgroups of primary food providers based on socio-economic position.

## Methods

This discrete choice experiment was prospectively registered by the Australian New Zealand Clinical Trials Registry (no. ACTRN12618001173280) and ethics approval obtained from the Flinders University Social and Behavioural Research Ethics Committee of South Australia (no. 8043). Reporting of this study was guided by the STROBE statement checklist (see Additional files 1, [32]). The study was undertaken online, July to September 2018, participants completed the study in one sitting of approximately 20 to 30 min. The online survey tool settings prevented participants from completing the study more than once.

Parents were eligible to take part in the study if they were the primary food provider for a three to seven-year-old child, residing in Australia and fluent in written English with access to the internet. Parents were excluded if they were under the age of 18 years.

Primary food providers were recruited through paid social media advertising (Facebook©), a study specific Facebook page, paper flyers, media and an online forum (BubHub). An additional recruitment strategy was employed to target parents residing in lower socio-economic areas, by contacting and requesting for parenting pages and playgroups in these areas to share the study details on their social media pages. An incentive of a chance to win one of ten $30 supermarket vouchers was offered to participants completing the study. Primary food providers provided consent online prior to completing the online survey.

Primary food providers were invited to complete an online survey (Qualtrics®) that contained five sections: 1) eligibility screening, 2) quasi revealed preferences and quality assurance items, 3) the discrete choice experiment, 4) characteristics of usual snack provision attributes, and 5) socio-demographics.

### Development of the discrete choice experiment survey instrument

The main component of the survey was the discrete choice experiment. The discrete choice experiment involved development of an elicitation task (including the choice condition, attributes and attribute levels, choice task), statistical experimental design and statistical modelling approach to understanding primary food providers’ snack provision preferences.

Two conditions were included in the discrete choice experiment: 1) a control scenario where participants were asked to make decisions for snacks provided assuming that only immediate family members were present (referred to as the ‘non-social’) and 2) a manipulated scenario which was a social condition, where participants were asked to assume they were making snack provision decisions as if immediate family members and family friends were present (‘social’ condition).

A list of potential attributes (i.e. characteristics or factors) was developed informed by qualitative [9, 11, 14, 15, 17, 20] and quantitative literature [10, 12, 13, 18, 19]. Attributes were selected for inclusion if they were commonly raised influences or reported to have significant associations with child intake, as well as researcher expertise. Physical resource attributes included cost, time and different types of food available. Type of food attribute was designed to measure availability of different types of food in the home. ‘Type of food’ attribute was also considered a measure of healthiness given the attribute levels were presented as everyday and sometimes foods (see Table 1 for examples). The definitions of everyday foods and sometimes foods were based on the Australian Dietary Guidelines [33]. Social support attributes included child resistance, co-parent support and family friends support. Attribute levels were informed by researchers who were primary food providers and aimed to reflect realistic levels, and ranged from two to three levels. See Table 1 for selected attributes and attribute levels.

**Table 1:** Design attributes and levels, and reference levels for analysis, and information provided to participants

Attribute	Attribute level	Excerpts from participant information
Cost of snack	Cheaper (reference level) More expensive	When considering the cost think about what you would think of as a: cheap and expensive snack as a reference point.
Time to prepare	Instant (reference level) Quick More time consuming	This will vary from instant which would be almost instant or ready to eat (such as taken straight from the fridge or pantry), quick so a few minutes (such as chopping, toasting or plating), or more time consuming which would be around 5 min or more (such as cooking, preparing multiple components).
Child’s likely response	Accepting (reference level) Resistant	Think about past experiences and how your child has responded to the food options you provide. For example, if it is a food your child does not prefer or may not feel like they might have been resistant to eating it.
Co-parent support	Supportive Unsupportive (reference level)	This refers to partners or co-parents. The opinion or role of these significant family members may vary between options from supportive (or consistent with you) to unsupportive (or undermining), depending on their values for food provision.
Family friend support	Supportive Unsupportive (reference level)	This refers to your (or your partners) friends with kids that you would spend time with as a family. As with family members the opinion or role of these family friends may vary between options from supportive (or consistent with you) to unsupportive (or undermining), depending on their values for food provision.
Type of food	Everyday foods Sometimes foods (reference level)	Everyday foods are the foods and drinks that we commonly refer to as the ‘five food group’ or ‘staple/core’ foods that we include in our meals and snacks every day. These foods come from the fruit, vegetable, dairy or alternatives, grain foods, and meat or alternatives food groups Sometimes foods are the foods and drinks that we commonly refer to as ‘extras’, ‘treats’ or ‘junk food’. Some examples include crisps, pastries, pizza, cake, sweet or savory biscuits, chocolate, muesli bars, and sugary drinks.

**Fig. 1:**
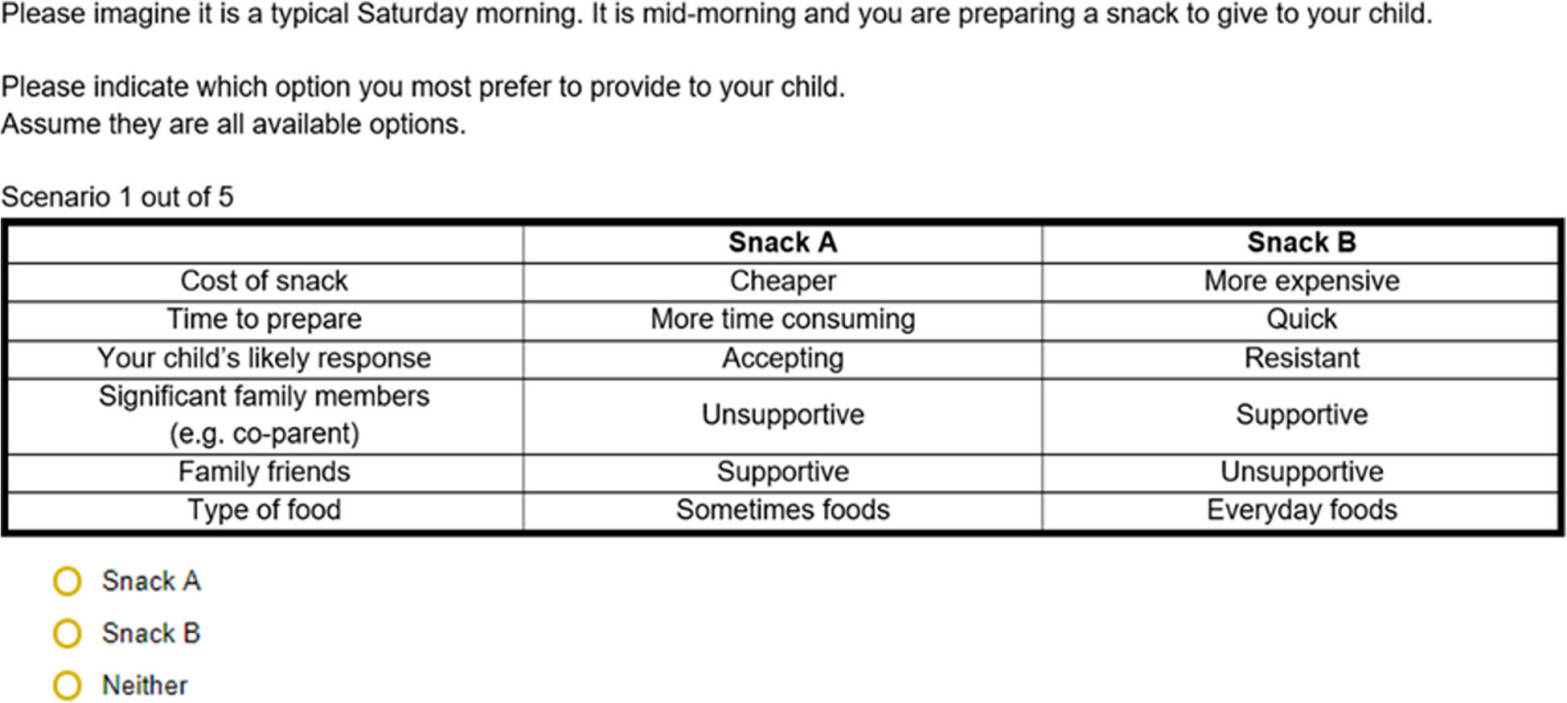
Example choice task presented to participants in the online discrete choice experiment

Participants were presented with five choice tasks, one at a time. In each choice task, participants were asked to select their preferred snack option from two alternatives of varying attribute levels (i.e. Snack A or Snack B) or neither (opt-out option) for a given condition scenario (non-social or social condition) (Fig. 1); “*It is mid-morning and you are preparing a snack to give your child. Please indicate which option you most prefer to provide to your child. Assume they are all available options*”. The opt-out option was included to enhance the external validity in the case where ‘neither’ of the options were appealing or appropriate to the participant. Each choice task presented all six attributes, however each alternative ‘snack’ varied in the attribute levels contained. The choice task is specifically designed to force participants to make trade-offs between attribute when deciding which alternative ‘snack’ they would provide to their child in the given scenario.

A statistical experimental approach was used to design the discrete choice experiment. The experimental design informs the choice tasks composition, which includes the attribute levels to be included in each alternative ‘snack’, and number of tasks to show a participant. An orthogonal main effects design was prepared using NGene (ChoiceMetrics 2018, version 1.2.0), based on the six attributes and corresponding levels (2 × 4 [time] × 2 × 2 × 2). This design was selected as the most appropriate to maximize the power of the design to detect significant relationships [28]. ‘Time to prepare’ attribute contained three levels, therefore the middle level was repeated in the design to ensure attribute balance across the design. The final design contained 20 unique choice tasks that were blocked into four blocks of five choice tasks. The same design was used for social and non-social conditions, resulting in ten choice tasks per participant. The discrete choice experiment was pilot tested with a convenience sample of colleagues, nutrition students and family to ensure the attributes and task were well understood, with minor revisions incorporated to the final discrete choice experiment.

Participants were randomized within the online survey to receive either the social or non-social condition first, then again randomized to one of four choice blocks. After completing the first condition block, participants completed a break activity (i.e. distraction) [34], prior to completing the remaining condition block. Attribute order was manually randomized within each choice task. An explanation of the choice task and a glossary was provided prior to the first task to assist participants in interpreting the attributes and levels, and to define a snack. See Table 1 for excerpts of information provided to participants. The online survey tool forced responses prior to continuation; therefore, completed records did not include missing responses.

### Socio-demographics and quality assurance– variables and measures

Participant socio-demographic items included age, gender, weight status, education level, employment status, ancestry and family structure. Child characteristics included age, gender and weight status. In addition, information was obtained about the household socio-economic position. Items were based on the questions used in the Australian Census [35] where possible. Participant self-reported weight and height were used to calculate body mass index (BMI) and classified to weight status categories [36]. Participant reported child weight and height were converted to BMI z-scores using the least mean squares method and classified to weight status categories [37–40]. Participants were also asked to indicate whether their child’s weight and/or height had been measured in past 6 months. Socio-economic position was determined by matching postcode to Socio-Economic Indexes for Areas (SEIFA) Index of Relative Socio-economic Advantage and Disadvantage (IRSAD) score and decile [41]. Subgroups were created for primary food providers living in lower SEIFA areas (IRSAD deciles of 1 to 5) and those living in higher SEIFA areas (IRSAD deciles of 6 to 10).

Prior to commencing the discrete choice experiment participants rated perceived barriers to their child eating a healthy diet and self-reported current examples of snacks provided in social and non-social conditions. Both items provided quasi revealed preference (referred to as real market) data, i.e. true provision, and are recommended to be compared with stated preference data obtained from discrete choice experiments to improve the external validity of the findings [42]. The perceived barriers item was adapted from a study by Slater and colleagues [12]. It is expected that perceived barriers to their child eating a healthy diet would be correlated with considerations for actual snack provision choice, therefore average rating of perceived barriers were compared with the results from the discrete choice experiments. A corresponding ranking would show support for the selection of attributes and validity of findings. Current snacks were assessed by two open text response items where participants reported common examples of snacks provided to their child in social and non-social occasions, these were phrased similarly to the scenarios. Each food and beverage item reported was coded as healthy (e.g. carrot sticks) or unhealthy (e.g. cake) guided by the Australian Bureau of Statistics [43] spreadsheet flagging foods classified as unhealthy foods. Unhealthy snack provision was calculated as a percentage of total reported usual snacks (healthy and unhealthy) and used as a crude measure for unhealthy food provision.

There are several general sample size guides for discrete choice experiments. Lancsar and Louviere [30] suggest 20 participants per block (e.g. 80 participants per condition). Johnson and Orme (2003, cited in de Bekker-Grob [44]) proposed a rule of thumb:: *N* > 500*c* / (*t* x *a*). Where *t* is the number of choice tasks (per participant), *a* is the number of alternatives and *c* is the largest number of attribute levels; therefore, a minimum sample of 150 per condition. Oversampling is further suggested to allow for selection of ‘neither’ option; based on prior research an estimate of 20% opt out was used to as an initial guide (i.e. requiring 30 extra participants). Study recruitment aimed for approximately 180 participants to meet all sample size estimates, as participants completed both a block of social and non-social condition.

### Statistics

Response data was imported into Microsoft Excel (2013, Microsoft Corporation, Redmond, WA, USA) and IBM SPSS Statistics (Version 25; SPSS Inc., Chicago, IL, USA) for cleaning. There were no missing data. Data were restructured to obtain stacked choice data with 15 cases per participant (5 choice tasks × 3 alternatives per task) for each condition. Choice task attributes levels were dummy coded per alternative (0 = level not presented; 1 = level presented). Total proportion of ‘neither’ choices was 10%, these responses were included in analyses, yet offered no insight into attribute importance.

Data were imported into Nlogit 6 (Student version, Econometric Software Inc., 2016) for multinomial logit model analyses of the discrete choice data per condition. Analysis of discrete choice experiment data is different to regression models, coefficients from choice models estimated on the choice data are interpreted as utility weights for each of the attributes to allow comparisons of the importance between attributes. Multinomial logit analyses are based on the assumptions of random utility theory, with the premise that respondents will choose the alternatives that will maximize their utility, including that people will trade off between attributes (i.e. compensatory decisions) [28, 45]. As utility is a latent construct, choices measured in the discrete choice experiment acted as indicators of utility [30]. Based on the assumption that the systematic part of utility is the sum of its parts, the utility weight can be determined for each component of utility (i.e. attribute) [30]. The terms utility weight and coefficient can be used interchangeably. The utility function equation was specified in Eq. 1, where V is the observable utility and in which the reference alternative was ‘neither’ and all attributes (β1 to β7) are dummy coded. In all models the constant was included as the utility for the ‘neither’ alternative. Reference levels were set as cheaper (cost), instant (time), child accepting, unsupportive co-parent, unsupportive family friend, and sometimes food (type of food) (Table 1), to interpret the first three attributes as disutility and the final three as utility.

1 $$ V=\beta j+\beta 1\  COST\ more\ expensive+\beta 2\  TIME\ quick+\beta 3\  TIME\ more\ time\ consuming+\beta 4\  CHILD\ resistant+\beta 5\  COPARENT\ supportive+\beta 6\  FRIEND\ supportive+\beta 7\  FOOD\ everyday\ foods $$

Models were estimated for the non-social and social conditions, as well as for subgroups of participants based on socio-economic position (Lower: deciles 1 to 5, versus Higher: deciles 6 to 10). It was hypothesized that in the context of social occasions type of food would not be important (i.e. non-significant) and support from family friends to be the most important influence in primary food providers' snack decision-making. To test for order effects on the uptake of the choice tasks, a model was estimated to also include a dummy coded variable for condition order (i.e. social versus non-social condition presented first). Model fit was determined by comparing model log-likelihood, likelihood ratio chi-square (indicator of goodness of fit), pseudo R^2^ (indicator of relative fit) and norm Akaike Information Criterion. Relative importance scores were calculated using the partial log-likelihood method recommended by Lancsar and colleagues [46] to measure the overall attribute effects relative to other attributes.

To account for potential heterogeneity in the sample, we accounted for observed preference heterogeneity by conducting subgroup analyses based on socio-economic position. Following Swait and Louviere [47], a scatterplot of the coefficients indicated that the relative scale parameter across subgroups was close to one (e.g. subgroup having similar error variance or consistency in their answers compared to another subgroup), indicating it was appropriate to test for statistical differences across subgroup results, this was tested by comparing the 95% confidence intervals between model results across conditions.

Choice data analysis outputs are presented as utility weights (i.e. coefficients), 95% confidence intervals, *p* value and relative importance scores. Significance was set at 0.05. For attributes with three levels (i.e., time to prepare), p value was calculated using the Wald test to account for the multiple attribute levels [28]. At completion of the discrete choice experiment participants selected attribute levels of their usual snack provision. Results were compared to the final model choice outputs to consider the external validity of the findings.

## Results

**Fig. 2:**
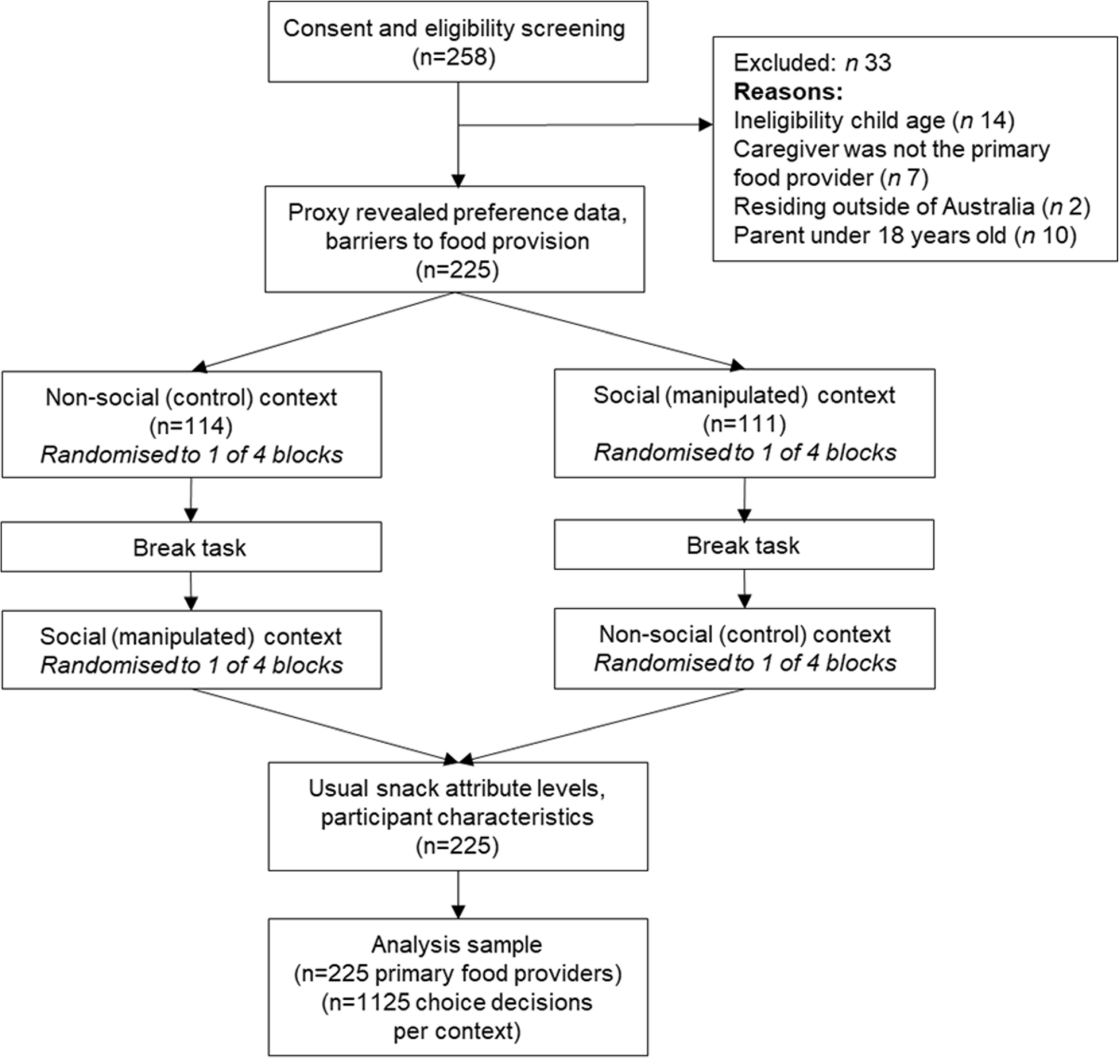
Flow chart of participants through the online survey and discrete choice experiment

Two-hundred and fifty-eight primary food providers commenced the online survey, of this 225 were eligible and completed the study (87%) (Fig. 2). Randomization achieved 114 participants completing the non-social condition first and 111 completing the social condition first. There was even representation of each of the choice task blocks (range 53 to 61 participants per block). The mean survey duration was 22 min (SD 16 min), indicating sufficient time to consider choice trade-offs. Table 2 presents descriptive characteristics of the primary food provider and child sample. Primary food providers were nearly exclusively mothers (99.6%), who were married or living as married (94.7%). Approximately half of the participant sample were employed part time (51.6%) and three quarters held a tertiary or postgraduate degree (72.5%). Children had a mean age of 5.2 years (SD 1.3) and approximately half were classified as within the healthy weight range (55.6%). Overall the attributes from the discrete choice experiment could be matched with participant rating of perceived barriers to healthy food provision (see Additional file 2 Supplemental Table 1). Usual attribute rating revealed primary food providers commonly provide snacks that co-parents (96.9%) and family friends (96.4%) are supportive of, are everyday foods (92.4%), their child is accepting of (92.0%), are quicker to prepare (84.4%), and cheaper (61.3%) (see Additional file 2 Supplemental Table 2).

**Table 2:** Descriptive characteristics of primary food providers and children (*n* = 225)

Characteristic	Parent	Characteristic	Child
Age, years (mean, SD)	35.3 (3.8)	Age, years (mean, SD)	5.2 (1.3)
Sex (%, count)		Sex (%, count)	
Male	0.4 (1)	Male	49.3 (111)
Female	99.6 (224)	Female	50.7 (114)
Weight status^a^ (%, count)		Weight status (%, count)	
Underweight	1.8 (4)	Underweight	13.9 (31)
Healthy weight	39.6 (86)	Healthy weight	55.6 (124)
Overweight	33.2 (72)	Overweight	15.2 (34)
Obesity	25.3 (55)	Obesity	15.2 (34)
Family structure (%, count)		Weight and/or height measured in past 6 months (%, count)	73.8 (166)
Couple with a child	13.8 (31)		
Couple with children	80.9 (182)		
One parent family with a child	0.9 (2)		
One parent family with children	1.8 (4)		
Other family type	2.7 (6)		
SEIFA^b^ Index of Relative Advantage and Disadvantage(%, count)		Frequency of social occasions in past week (median, IQR)	5 (4)
Lower (deciles 1 to 5)	40.4 (91)	Frequency of select celebratory occasions in past week (median, IQR)	0 (1)
Higher (deciles 6 to 10)	59.1 (133)		
Parent education (%, count)			
Completed high school or less	6.7 (15)		
Tech or trade	20.9 (47)		
Tertiary degree	35.6 (80)		
Postgraduate degree	36.9 (83)		
Parent employment (%, count)			
Employed full time	18.2 (41)		
Employed part time	51.6 (116)		
Not working / homemaker	30.2 (68)		
Ancestry^c^ (%, count)			
Australian	48.0 (108)		
English	45.8 (103)		
Other	26.6 (60)		
Scottish	14.2 (32)		
Irish	13.3 (30)		
German	6.2 (14)		
Italian	5.3 (12)		

^a^Missing anthropometric responses for primary food providers (*n* = 8) and for children (*n* = 2)

^b^SEIFA, Socio-Economic Indexes for Areas; a lower value is reflective of greater disadvantage. Missing SEIFA (*n* = 1)

^c^Participants could select up to two ancestries, therefore percentages exceed 100

All participants completed five choice tasks per condition, providing 1125 choice observations per condition for analyses. The indicator for condition order was significant (non-social utility weight = − 0.751, *p* < 0.001; social − 0.544,* p *< 0.001) suggesting there was an average order effect on the overall uptake of the choice task, but there were no differences in attribute importance. Presented analyses controlled for order effects. Five of the six attributes were found to significantly influence primary food providers’ snack provision decision-making: type of food, child resistance, co-parent support, friends support and cost (Table 3). The time to prepare attribute was not significant. This was consistent in both social and non-social conditions. Utility weights could be directly compared between conditions as scale parameter was near one (0.95). Negative utility weights indicated that primary food providers preferred snack options that were lower in cost, time and elicited less child resistance. Positive direction of the remaining attributes indicated that primary food providers preferred snack options where ‘everyday foods’ were available, and co-parents and friends were supportive of the options. Relative importance scores indicated similar importance for attributes in both non-social (1: type of food; 2: child resistance; 3: co-parent support) and social conditions (1: child resistance; 2: type of food; 3: co-parent support). Examining utility weights in non-social conditions revealed type of food attribute was 20% more influential than the disutility for child resistance. Support from co-parents had double the influence of support from friends in the non-social condition (utility weight 0.998, 95%CI 0.774 to 1.223, *p* < 0.001 vs 0.448, 0.220 to 0.675, *p* < 0.001). Within the social condition, co-parent support was only 25% more influential than support from friends (utility weight 1.077, 95%CI 0.855 to 1.298, *p* < 0.001 vs 0.794, 95%CI 0.575 to 1.014, *p* < 0.001). When comparing 95% confidence intervals for social condition utility weights with non-social utility weights, it was suggested there may be a difference in support from friends and type of food attributes. However, there were no convincing statistical difference between remaining attributes, so general comparisons were made. The difference in the influence of type of food and child resistance was smaller in social conditions, than for non-social condition.

**Table 3:** Non-social and social occasions multinomial logit model analysis results^a^

Attributes	Non-social condition		Social condition	
	Utility weight (95%CI)	Relative importance score	Utility weight (95%CI)	Relative importance score
**Cost**				
Cheaper (reference level)	−0.333 (−0.586 to −0.081)*	5	−0.320 (−0.552 to −0.087)*	5
More expensive				
**Time**^b^				
Instant (reference level)	0.115 (−0.227 to 0.458)	6	−0.077 (− 0.402 to 0.248)	6
Quick				
More time consuming	−0.096 (− 0.428 to 0.236)		− 0.162 (− 0.491 to 0.166)	
**Child’s likely response**				
Accepting (reference level)	−1.624 (−1.851 to −1.398)**	2	−1.506 (−1.722 to −1.291)**	1
Resistant				
**Support from co-parent**				
Supportive	0.998 (0.774 to 1.223)**	3	1.077 (0.855 to 1.298)**	3
Unsupportive (reference level)				
**Support from friends**				
Supportive	0.448 (0.220 to 0.675)**	4	0.794 (0.575 to 1.014)**	4
Unsupportive (reference level)				
**Type of food**				
Everyday foods	1.944 (1.685 to 2.202)**	1	1.384 (1.154 to 1.614)**	2
Sometimes foods (reference level)				
**Neither alternative**	−1.256 (−1.778 to −0.734)**		−1.066 (− 1.556 to −0.575)**	
**Model fit statistics**				
Log likelihood of model	− 756.18		− 827.29	
Log likelihood of model without predictors	− 1204.62		− 1207.44	
Likelihood ratio X^2^	896.88		760.30	
Norm. Akaike information criterion	1.360		1.487	
Pseudo R^2^	0.372		0.315	

^a^No. of respondents *n* = 225, No. of observations *n* = 1125. Adjusted for condition order effects

^b^Wald test p value presented for time attribute (quick + more time consuming = 0)

* *p* < 0.05, ** *p* < 0.001

Abbreviations: *X*^*2*^ chi-square; *95%CI* 95% confidence interval

### Examination of relative importance within socio-economic subgroups

**Fig. 3:**
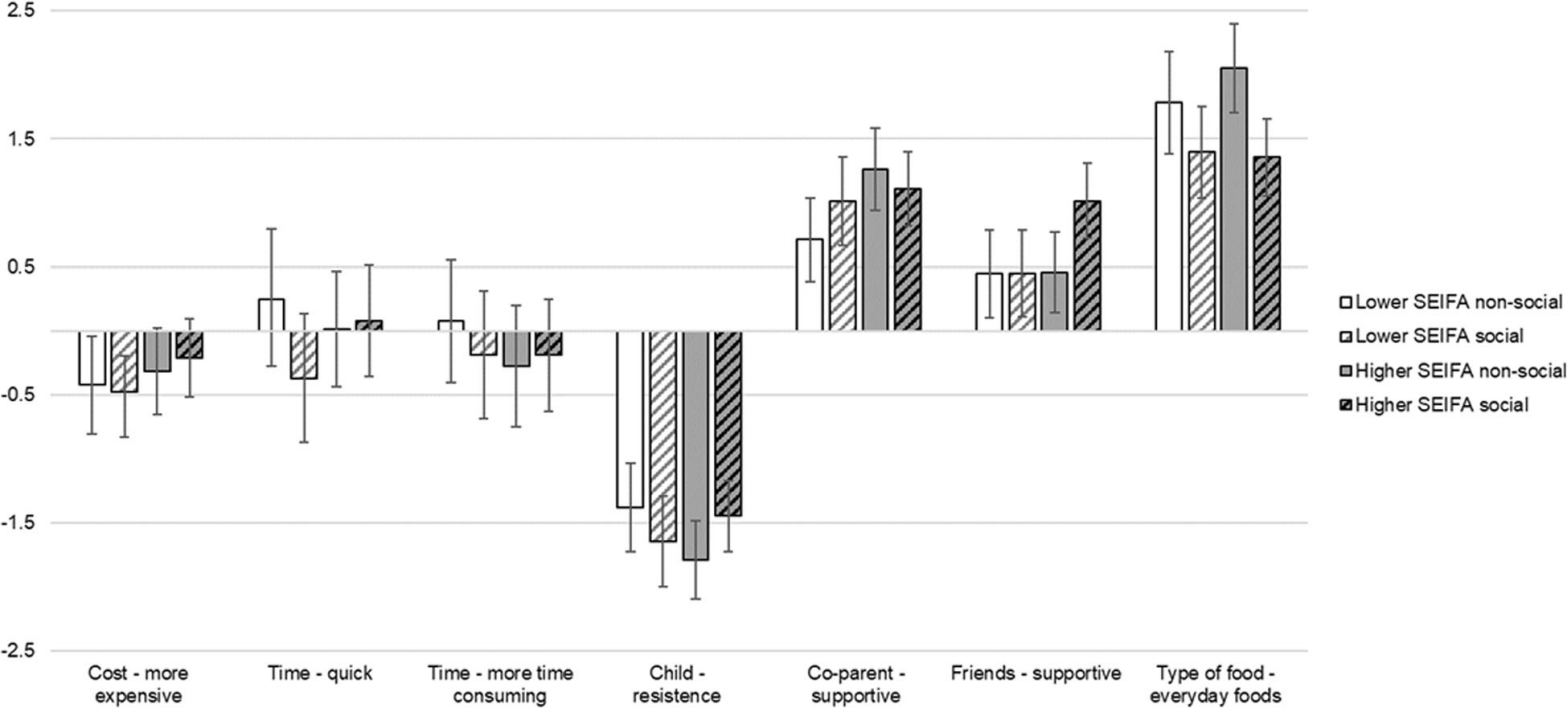
Utility weights from subgroup analysis by socio-economic position for non-social and social occasions. White shading columns represent lower socio-economic position subgroup (*n* = 91, 455 choice observations), grey shading columns represent the higher socio-economic position subgroup (*n* = 133, 665 choice observations). Block color represents the non-social condition, diagonal stripes represent the social conditions. Error bars represent 95%CI, error bars that cross zero represent non-significant influences

Figure 3 presents utility weights by socio-economic subgroups. In the sample of participants living in lower SEIFA (*n* = 91) the relative importance of attributes were interpreted in a similar pattern as the whole sample with type of food and child’s likely response the most important attributes, in non-social and social conditions, respectively. The key difference being that in social conditions cost was ranked of higher importance than support from friends. In the group living in higher SEIFA areas (*n* = 133), attributes were also found to have the same relative importance scores, however the cost attribute did not have a significant influence on primary food provider decision-making in either condition.

## Discussion

This study, to the best of our knowledge, is the first to determine the relative importance of physical resources and social supports in primary food providers’ snack provision decision-making, in both social and non-social conditions. Type of food was identified as the most important influence in primary food providers’ decision-making in the non-social condition. Child resistance ranked of highest importance in the social condition. Comparison of snack decision making by social condition, and subgroups revealed limited differences in the selected scenario. This study extends our knowledge of the influences on primary food providers’ snack provision and provides statistically determined priorities for future initiatives to target the types of food available at home, child resistance and co-parent support as initial intervention targets.

There was little variation in the relative importance of attributes when comparing social conditions or socio-economic subgroups. Type of food, child resistance and co-parent support remained the three most important attributes in all conditions and subgroups, despite the order of the top two attributes differing by social condition. The findings suggest that when forced to make trade-offs, in the presence of family friends (i.e. social condition), primary food providers place a greater relative importance on snacks that children are more accepting of and slightly lower importance on healthier types of food. It should be noted that while the type of food attribute was designed to reflect the availability of healthy or unhealthy foods, this attribute may also encompass participants food provision preferences, or knowledge and attitudes towards a healthy diet. Findings somewhat supported our hypothesis that influences on primary food providers’ decision-making, specifically type of food and support from family friends, would differ in social vs non-social occasions, albeit differences were relatively small. We did not ask participant about whether their friends food provision philosophies align with their own, if they have common beliefs this may explain why support from family friends was not of greater relative importance in social occasions. Past literature supports this finding with child resistance and preferences identified as challenges for primary food providers’ food provision, in many cases to avoid conflict and maintain a calm environment [11, 12, 14, 15, 17, 19]. Co-parent support, across the sample, was consistently ranked third in terms of relative importance, building evidence [48, 49] of the importance of intervening on the co-parenting relationship or including co-parents in interventions.

There were slight subgroup differences in the significance of lower ranked attributes. Cost was not found to be a statistically significant influence in the subgroup of primary food providers living in higher socio-economic areas, in both social and non-social conditions, but was significant for primary food providers living in lower socio-economic areas in both conditions. Our finding is consistent with a discrete choice experiment study of adult’s meal choice, finding cost of higher relative importance in most disadvantaged subgroups [50]. Regardless of this difference, findings were consistent for the primary influences for all families, namely type of food, child resistance, and co-parent support.

Our findings suggest interventions should consider a whole of family approach by reducing availability of unhealthy snacks in the home and engaging co-parents and children to mitigate resistance. Designing interventions to address these attributes will enhance primary food providers physical and social opportunity—defined as “all the factors that lie outside the individual that make the behavior possible or prompt it” [8](p. 4 of 11). Health psychology experts propose that for behavior change to occur parents need to have the capability (i.e. knowledge, skills), opportunity and motivation [8]. Our prior critique of past interventions seeking to reduce parental provision of unhealthy foods, found very few interventions targeted changes in parents’ opportunity, instead focusing heavily on parents’ capability or motivation [51]. Only one study reviewed targeted physical opportunity by addressing food access and availability within the home [52]. Thus, combined these findings suggest that current interventions fail to support primary food providers regarding important physical resource and social support influences of their food provision choices. In addition, it is important to acknowledge the interconnections between the top ranked attributes; children’s preferences for different types foods may predict their likely response to being offered certain snacks and influence the types of foods that are brought into the home. When tailoring intervention content to families living in lower socio-economic areas, the cost of snacks may also need to be addressed, but in the context of type of food available, child resistance and co-parent support.

Our study capitalized on the strengths of the discrete choice experiment design. Discrete choice experiments in the health field have largely been used to explore healthcare products and programs [30, 53]. There are few applications of this method in the nutrition field [31, 54, 55], with those available commonly exploring characteristics of front-of-packet labeling [56, 57]. We have made a unique contribution to this literature by exploring food provision decision-making. In addition, this is the first study to include two conditions—social and non-social—using the same design and compare results across the discrete choice experiments in any field. We designed the experiment to allowed direct comparison of both physical resources and social supports in the one sample, which has not been done comprehensively before and allowed us to determine the relative importance across these commonly reported barriers. The discrete choice experiment method also attenuates social desirability bias through repeated hypothetical choice tasks and by forcing trade-offs [28]. This method, in contrast to traditional survey or interview approaches, avoids reliance on participants to self-report barriers, where social desirability may be more prominent. However, there is still potential that social desirability may have been present with the type of food attribute levels reflecting ‘everyday’ or ‘sometimes’ foods, hence may have been perceived as a healthiness measure. Finally, we used randomization to mitigate any bias from order effects, and attempted to account for preference and scale heterogeneity.

As is the case with discrete choice experiments, hypothetical bias [29] was a limitation as participants were not actually providing snacks to their child. The choice tasks in this study attempted to reduce the hypothetical bias by closely mimicking food provision for primary food providers. Secondly, there were limitations relating to the design of the discrete choice experiment, specifically the use of unlabeled alternatives and end-point attribute levels. The use of unlabeled snacks/non-branded rather than specific food items, may have added to the cognitive burden of the choice tasks as participants had to imagine the type of snacks. However, unlabeled snacks were selected to better accommodate for differences in common snacks across families and consider the social support attributes. While the number of attributes and levels were consistent with standard practice to reduce cognitive burden of participants in the study, primarily end-point levels were used for attributes (e.g. supportive vs non-supportive), therefore may not have captured the variation that could be measured with greater number of attribute levels. In addition, the ‘type of food’ attribute may have encompassed more than availability of healthy or unhealthy foods and captured participants food provision preferences and value placed on healthiness. Finally, while we did have a relatively even representation of primary food providers residing in areas of low, moderate and high socio-economic position, the sample had a greater number of participants with high education attainment—76% with a tertiary degree or higher versus 36% of Australian mothers [58]. There was also underrepresentation of fathers, however this is not surprising due to the gendered differences in parents’ employment and division of household labor in Australia often resulting in mothers taking on the role of primary food provider [59].

There are several additional implications for future research. Our findings signal an opportunity to further explore the role of cost and time, including if their importance differs in snacks compared to meals, and when using multiple, individual focused indictors of socio-economic position (e.g. income, parental education). The role of unhealthy foods in social occasions also warrants further exploration to inform targeted strategies in future interventions given the frequency of social occasions reported in our sample. Finally, discrete choice experiment methods offer opportunities to consider other food provision conditions and attributes, such as weekdays or out of home intake with additional attribute levels more sensitive in measuring utility.

## Conclusions

Type of food, child resistance and co-parent support were found to be of greatest relative importance in primary food providers’ snack provision decision-making. Findings provide additional support for prior observational research, strengthened by the different methodological design and by determining the relative importance when considering physical resources and social supports in the one sample of primary food providers. Future interventions should prioritize consideration of the types of food available at home, child resistance and co-parent support to assist families in reducing unhealthy food intake in snack occasions.

## Supplementary Information

**Additional file 1.** Reporting Checklist.

**Additional file 2.** Quasi revealed preference data.

## Data Availability

The datasets used and/or analysed during the current study are available from the corresponding author on reasonable request.

## Abbreviations

BMI: Body mass index
IRSAD: Index of Relative Socio-economic Advantage and Disadvantage
SEIFA: Socio-Economic Indexes for Areas
