# Supplementary material for: Understanding the influence of physical resources and social supports on primary food providers’ snack food provision: a discrete choice experiment
Source: Int J Behav Nutr Phys Act. 2020 Nov 30;17:155. doi: 10.1186/s12966-020-01062-y (PMC7706064; doi:10.1186/s12966-020-01062-y)
Supplement: Supplementary file 1 — Additional file 1. Reporting Checklist. [file 12966_2020_1062_MOESM1_ESM.docx]

**Additional file 1: Reporting Checklist**

STROBE Statement: Checklist of items that should be included in reports of ***cross-sectional studies***

| **Section / topic** | **Item No** | **Recommendation** | **Page** |
| --- | --- | --- | --- |
| **Title and abstract** | 1 | (a) Indicate the study’s design with a commonly used term in the title or the abstract | 1 |
|  |  | (b) Provide in the abstract an informative and balanced summary of what was done and what was found | 3 |
| **Introduction** | | |  |
| Background/rationale | 2 | Explain the scientific background and rationale for the investigation being reported | 5-6 |
| Objectives | 3 | State specific objectives, including any prespecified hypotheses | 6 |
| **Methods** | | |  |
| Study design | 4 | Present key elements of study design early in the paper | 6 |
| Setting | 5 | Describe the setting, locations, and relevant dates, including periods of recruitment, exposure, follow-up, and data collection | 6-7 |
| Participants | 6 | (a) Give the eligibility criteria, and the sources and methods of selection of participants | 6-7 |
| Variables | 7 | Clearly define all outcomes, exposures, predictors, potential confounders, and effect modifiers. Give diagnostic criteria, if applicable | 7-10 |
| Data sources/ measurement | 8 | For each variable of interest, give sources of data and details of methods of assessment (measurement). Describe comparability of assessment methods if there is more than one group | 7-10 |
| Bias | 9 | Describe any efforts to address potential sources of bias | 8-9 |
| Study size | 10 | Explain how the study size was arrived at | 10 |
| Quantitative variables | 11 | Explain how quantitative variables were handled in the analyses. If applicable, describe which groupings were chosen and why | 11 |
| Statistical methods | 12 | (a) Describe all statistical methods, including those used to control for confounding | 11-12 |
|  |  | (b) Describe any methods used to examine subgroups and interactions | 11 |
|  |  | (c) Explain how missing data were addressed | 11 |
|  |  | (d) If applicable, describe analytical methods taking account of sampling strategy | n/a |
|  |  | (e) Describe any sensitivity analyses | 12 |

STROBE Statement: Checklist of items that should be included in reports of ***cross-sectional studies*** (cont.)

| **Section / topic** | **Item No** | **Recommendation** | **Page** |
| --- | --- | --- | --- |
| **Results** | | |  |
| Participants | 13 | (a) Report numbers of individuals at each stage of study—e.g. numbers potentially eligible, examined for eligibility, confirmed eligible, included in the study, completing follow-up, and analysed | 12-113, Figure 2 |
|  |  | (b) Give reasons for non-participation at each stage | Figure 2 |
|  |  | (c) Consider use of a flow diagram | Figure 2 |
| Descriptive data | 14 | (a) Give characteristics of study participants (e.g. demographic, clinical, social) and information on exposures and potential confounders | 13, Table 2 |
|  |  | (b) Indicate number of participants with missing data for each variable of interest | n/a |
| Outcome data | 15 | Report numbers of outcome events or summary measures | 13 |
| Main results | 16 | (a) Give unadjusted estimates and, if applicable, confounder-adjusted estimates and their precision (e.g., 95% confidence interval). Make clear which confounders were adjusted for and why they were included | 13-14, Table 3 |
|  |  | (b) Report category boundaries when continuous variables were categorized | n/a |
|  |  | (c) If relevant, consider translating estimates of relative risk into absolute risk for a meaningful time period | n/a |
| Other analyses | 17 | Report other analyses done—e.g. analyses of subgroups and interactions, and sensitivity analyses | 14-15, Figure 3 |
| **Discussion** | | |  |
| Key results | 18 | Summarise key results with reference to study objectives | 15-17 |
| Limitations | 19 | Discuss limitations of the study, taking into account sources of potential bias or imprecision. Discuss both direction and magnitude of any potential bias | 18 |
| Interpretation | 20 | Give a cautious overall interpretation of results considering objectives, limitations, multiplicity of analyses, results from similar studies, and other relevant evidence | 15-18 |
| Generalisability | 21 | Discuss the generalisability (external validity) of the study results | 18 |
| **Other information** | | |  |
| Funding | 22 | Give the source of funding and the role of the funders for the present study and, if applicable, for the original study on which the present article is based | Acknowledgements |
