# Supplementary material for: Understanding the influence of physical resources and social supports on primary food providers’ snack food provision: a discrete choice experiment
Source: Int J Behav Nutr Phys Act. 2020 Nov 30;17:155. doi: 10.1186/s12966-020-01062-y (PMC7706064; doi:10.1186/s12966-020-01062-y)
Supplement: Supplementary file 2 — Additional file 2. Quasi revealed preference data. [file 12966_2020_1062_MOESM2_ESM.docx]

**Additional file 2: Quasi revealed preference data**

***Comparisons of relative importance scores to self-rated perceived barriers***

Parent rating of perceived barriers to healthy food provision was used in a confirmatory analysis to assess the suitability and construct validity of the discrete choice experiment design attributes and findings (**Supplemental Table 1**). Overall the attributes from the discrete choice experiment could be matched with barriers that spanned a mix of high (time and child resistance), medium (cost, time and co-parent support) and low rated barriers (type of food and friend support). Relative importance scores for attributes from the discrete choice analysis were compared with average rating of perceived barriers to food provision. Child resistance was the only attribute that aligned in terms of ranking with the reported barrier, both methods ranked child resistance as second most important consideration. Cost and time were ranked highly from parent report barriers, however obtained the lowest relative importance scores within the discrete choice experiment.

**Supplemental Table 1**: Percent of parent ratings of factors that make providing a healthy diet difficult^1^, matched to attributes

|  | **Parent rating barriers** | | **Discrete choice experiment relative importance scores** | | |
| --- | --- | --- | --- | --- | --- |
| Factors perceived as making providing a healthy diet difficult | Makes it a little or very difficult^2^ | Does not make it difficult | Corresponding attribute | Non-social | Social |
| **1. Busy lifestyle** | **83.1** | **16.9** | **~Time** | **6** | **6** |
| **2. Child resistance** | **76.0** | **24.0** | **Child resistance** | **2** | **1** |
| 3. Grandparents undermining provision | 57.3 | 42.7 | - |  |  |
| **4. High cost of healthy food** | **49.8** | **50.2** | **Cost** | **5** | **5** |
| **5. Effort required to provide healthy** | **46.2** | **53.8** | **~Time** | **6** | **6** |
| 6. Influences of child's peers | 45.3 | 54.7 | - |  |  |
| **7. Co-parent undermining provision** | **43.6** | **56.4** | **Co-parent support** | **3** | **3** |
| 8. Food advertising | 41.3 | 58.7 | - |  |  |
| 9. Ability to set rules and stick to them | 32.4 | 67.6 | - |  |  |
| 10. Knowing how to get child to eat healthy foods | 31.1 | 68.9 | - |  |  |
| **11. Healthy food availability** | **24.4** | **75.6** | **~Type of food** | **1** | **2** |
| **12. Expectations from my friends about what to provide** | **20.9** | **79.1** | **Friend support** | **4** | **4** |
| 13. Knowing what foods to provide / allow | 20.4 | 79.6 | - |  |  |

^1^ Item adapted from Slater and colleagues(10)

^2^ Response options 1) makes very difficult and 2) makes it a little difficult are presented as combined response.

**Bolded** factors correspond to current attributes in the discrete choice experiment

***Usual snack provision***

Parents reported common snacks provided in both social and non-social occasions. This data were used as a proxy measure for revealed preference data regarding parents snack provision. Snack items were categorized as healthy or unhealthy based on the Australian Dietary Guidelines discretionary choices definition, and the proportion of unhealthy food snacks was calculated. In the non-social context the mean percentage of unhealthy food snacks was 29.0% (SD 23.2%), compared with 45.9% (SD 29.9%) in the social context. Usual attribute rating revealed parents commonly provide snacks that are cheaper (61.3% of parents), quick to prepare (84.4%), child is accepting of (92.0%), co-parents (96.9%) and family friends (96.4%) are supportive of, and that are everyday foods (92.4%) (**Supplemental Table 2**).

**Supplemental Table 2**: Parent reported usual^1^ snack attribute levels

| **Attribute** | **Attribute levels** | **% (count)** |
| --- | --- | --- |
| Cost | **Cheaper** | **61.3 (138)** |
|  | More expensive | 38.7 (87) |
| Time to prepare | Instant | 6.2 (14) |
|  | **Quick** | **84.4 (190)** |
|  | More time consuming | 9.3 (21) |
| Child's likely response | **Accepting** | **92.0 (207)** |
|  | Resistant | 8.0 (18) |
| Co-parent support | **Supportive** | **96.9 (218)** |
|  | Unsupportive | 3.1 (7) |
| Friends support | **Supportive** | **96.4 (217)** |
|  | Unsupportive | 3.6 (8) |
| Type of food | **Everyday foods** | **92.4 (208)** |
|  | Sometimes foods | 7.6 (17) |

^1^ Ratings were based on any context

**Bolded** text signals higher frequency
